# Improvement of Navigation and Representation in Virtual Reality after Prism Adaptation in Neglect Patients

**DOI:** 10.3389/fpsyg.2017.02019

**Published:** 2017-11-20

**Authors:** Bertrand Glize, Marine Lunven, Yves Rossetti, Patrice Revol, Sophie Jacquin-Courtois, Evelyne Klinger, Pierre-Alain Joseph, Gilles Rode

**Affiliations:** ^1^Centre de Recherche en Neurosciences de Lyon, ImpAct Team, Institut National de la Santé et de la Recherche Médicale U1028, Centre National de la Recherche Scientifique UMR5292, Université Claude Bernard Lyon 1, Lyon, France; ^2^Service de Médecine Physique et de Réadaptation, Hôpital Henry Gabrielle, Hospices Civils de Lyon, Lyon, France; ^3^EA4136, Service de Médecine Physique et de Réadaptation, CHU de Bordeaux, Université de Bordeaux, Bordeaux, France; ^4^Groupe Hospitalier Pitié-Salpêtrière, Institut National de la Santé et de la Recherche Médicale U1127, UPMC-Paris 6, Centre National de la Recherche Scientifique UMR 7225, Institut du Cerveau et de la Moelle épinière ICM, Paris, France; ^5^ESIEA, Digital Interactions Health and Disability, Laval, France; ^6^Institut Fédératif de Recherche sur le HANDICAP, Paris, France

**Keywords:** spatial neglect, prism adaptation, virtual reality, navigation, rehabilitation, spatial representation

## Abstract

Prism adaptation (PA) is responsible for an expansion of sensori-motor after-effects to cognitive domains for patients with spatial neglect. One important question is whether the cognitive after-effects induced by PA may also concern higher aspects of spatial cognition, such as navigation and topographic memory, which are critical in everyday life. The aim of this study was to assess whether multiple sessions of right PA can affect navigation and topographic memory. Seven right brain-damaged (RBD) patients with chronic neglect were included. We used a virtual supermarket named VAP-S which is an original paradigm, similar to the “shopping list test” during which patients had to purchase items from a list of eight products. Furthermore, in order to assess generalization of PA effects on constructing a spatial map from virtual information, each participant was then asked to draw the map of the virtual supermarket from memory. Regarding navigation performance, significant results were obtained: session duration reduction, fewer numbers of pauses and omissions, more items purchased on the left side and more items purchased over all. A long-lasting effect was noted, up to one month after PA. The representational task performance was also significantly increased for map drawing, with a reduction of the right shift of the symmetry axis of the map, more items drawn on the left side of maps and over all, and more items correctly located on the map. Some of these effects lasted for at least 7 days. These results suggest an expansion of PA benefit to a virtual environment. Crucially, the cognitive benefits induced by PA were noted for complex spatial cognition tasks required in everyday life such as navigation and topographic memory and this improvement was maintained for up to 1 month.

## Introduction

Spatial neglect is one of the most intriguing deficits of spatial cognition. It is defined as an inability to detect, respond to, or orient one's attention toward stimuli presented or represented on the contralateral side of a brain damage, most frequently in the right hemisphere (Rode et al., 2016). The syndrome worsens the severity of associated motor and sensory deficits and constitutes a factor for poor functional prognosis, decreasing the ability to benefit from treatment (Denes et al., 1982; Fullerton et al., 1986; Halligan et al., 1989; Cherney et al., 2001; Di Monaco et al., 2011). Several rehabilitation methods have been proposed in view of reducing the behavioral bias on the side of the brain damage and the awareness deficit characterizing the contralateral hemi-space of spatial neglect (Rode et al., 2001a; Luauté et al., 2006).

Rehabilitation through prism adaptation (PA) is one of the most widely used and studied methods, and also one of the most effective (Jacquin-Courtois et al., 2013). It consists in using the systematic leftward shift of visuomotor and proprioceptive responses induced during an active exposure to a rightward optical deviation of the visual field. This method re-orients behavior of neglect patients toward the neglected side and produces a reduction of their deficits: visual neglect (Rossetti et al., 1998; Làdavas et al., 2001), space and object based neglect, sensory neglect (Dijkerman et al., 2003; Maravita et al., 2003), auditory neglect (Jacquin-Courtois et al., 2010), spatial dyslexia (Farnè et al., 2002), spatial dysgraphia, visuoconstructive disorders (Rode et al., 2006), and/or postural imbalance (Tilikete et al., 2001; Hugues et al., 2015). Visuo-manual after-effects of PA thus involve a regression of a wide range of perceptual, cognitive, and motor manifestations of spatial neglect as well as improvements of daily life activities such as writing, reading, posture, and wheelchair driving (see review Jacquin-Courtois et al., 2013). Negative results have also been reported by other studies (Ferber et al., 2003; Rousseaux et al., 2006; Nys et al., 2008; Turton et al., 2010; Rode et al., 2015). These differences could be explained by differences in the PA procedure, topography of the brain damage, and subtypes of spatial neglect assessed after PA. For example, positive effects were reported for peripersonal neglect, but not for personal neglect after PA (Priftis et al., 2013).

Expansion of the sensorimotor after-effects of PA to cognitive domains was reported in mental imagery tasks free from manual responses and overt visual scanning. This point is critical as manual actions and vision are crucially involved in the PA procedure. An improvement of representational neglect was thus demonstrated in two distinct tasks: explicitly spatial - the map of France, (Rode et al., 1998, 2001b)—and implicitly spatial - mental number bisection, (Rossetti et al., 2004; Aiello et al., 2013)—after a single session of PA. In the first case, an entire exploration of the map was possible after adaptation with evocation of cities from the western half. In the second case, the rightward bias found for mental number bisection was dramatically reduced (Rossetti et al., 2004, 2005). These findings demonstrate that the re-orientation of attention toward the neglected side induced by exposure to a rightward optical deviation of the visual field concerns not only the left part of physical space (extracorporeal and corporeal), but also the left part of imaginary space. This re-orientation of covert attention shows that the recalibration of visuomotor transformations induced by active prism exposure expands to higher-level supramodal representations of space (Striemer and Danckert, 2007; Nijboer et al., 2008; Schindler et al., 2009).

One interesting question that remains to be investigated is whether cognitive after-effects induced by PA also can expand to other aspects of spatial cognition such as navigation and topographic memory in a virtual environment (Ogourtsova et al., 2017). Previous studies found that PA involves an improvement of wheel-chair driving or walking in neglect patients, which in turn leads to an improvement of navigation and a reduction of disability (Jacquin-Courtois et al., 2008; Watanabe and Amimoto, 2010; Rabuffetti et al., 2013).

Hence, the present study aimed to assess whether PA can reduce the rightward attentional bias in a virtual reality task and improve navigation and topographic memory in patients with chronic neglect.

## Methods

**Figure 1:**
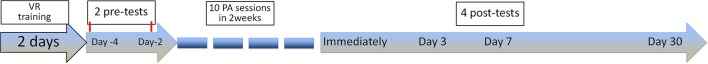
Experimental procedure. The procedure consisted of two pre-tests (at day−4 and day−2 prior to prism exposure) and four post-tests (immediately after prism removal; 0 h, and at 3, 7, and 30 days later). Each pre- or post-test consisted of subjective straight-ahead pointing test, VAP-S task, and drawings from memory task. VR, Virtual reality; PA, Prism adaptation.

This study was carried out in the neurological rehabilitation department of the Lyon teaching hospital (*Hospices Civils de Lyon*) from November 2012 to April 2013 and was approved by the Research Ethic Boards of the University Hospital of Lyon (CPP Sud-Est II). In order to meet the requirements of prior proof of principle, we included a small sample of patients and healthy controls. Figure 1 summarizes the procedure. The VR task and pencil-and-paper tests were performed at six time-points for the patients and the controls: two pre-tests at day−4 and day−2 prior to prism exposure, and four post-tests after intervention upon prism removal (0 h post-test), and at 3, 7, and 30 days thereafter.

### Participants

Seven right-handed (confirmed by Edinburgh Handedness Inventory (Oldfield, 1971) right brain-damaged (RBD) patients (six men, one woman; 59–70 years old; mean = 65.5; Standard Deviation (*SD*) = 4.1) with neglect following a first stroke were recruited in the chronic phase (mean delay post-stroke = 63 months; *SD* = 43). All patients had a visual neglect, assessed by five paper-and-pencil tests, including a line cancelation test (Albert, 1973), with measure of omissions on left (/18), center (/4) and right half (/18), the bells cancelation test (Gauthier et al., 1989) with measure of omissions on left (/15) and right half (/15), and a line bisection task (Schenkenberg et al., 1980). This latter includes 18 lines organized in three sets of six lines, so that one set lays primarily on the left side of the page, one in the center, and one on the right side. Subjects received instructions to mark the center of each line with a soft pen, without skipping any. The length of the left side of the line (i.e., from the left end of the line to the subject's mark) was measured to the nearest millimeter. That measurement was converted to a standardized score (percentage deviation), using the following formula: measured left half—objective half/objective half × 100. A positive sign indicates a deviation toward the right side, and a negative sign corresponds to a deviation toward the left side. The fourth test was a copy drawing task (Gainotti et al., 1972) with number of omissions (/10). The fifth test was a free drawing of a daisy from memory, with measurement of the number of petals on the left side or the right side considering the symmetry axis of the daisy (Rode et al., 2001b) (see Table 1). In order to assess improvement of neglect symptoms, these paper-and-pencil tests were performed at the inclusion and at each time-point before and after PA (i.e., the two pre-tests and the four post-tests), strictly following the same procedure. Patients did not suffer from post-stroke cognitive impairment [mean Mini Mental State Examination (MMSE) = 28; *SD* = 1.7], previous neurological or psychiatric disorders. No patient presented inability to use a VR device or to understand instructions. Characteristics of patients are given in Table 1. All patients had activity limitations (mean Barthel index = 70; *SD* = 15).

**Table 1:** Patient characteristics at inclusion.

**Patient**	**Age/sex**	**MD**	**SSD**	**LHH**	**OCD**	**LCT**	**BCT**	**LBT**	**CDT**	**DDFM**	**Etiology**	**Lesion volume (cm^3^)**	**White matter lesion site**
P1	70/M	3	3	P	1	18 L/0 R	14 L/1 R	23%	5	0 L/3 R	hem.	25.93	NA^a^
P2	59/F	1	0	P	1	18 L/0 R	15 L/2 R	−3%	5	2 L/2 R	isch.	211.60	IFOF, ILF, (AF)
P3	58/M	2	2	A	1	0 L/0 R	8 L/2 R	0.5%	1	6 L/6 R	isch.	226.95	SLF2, SLF3, IFOF, ILF, AF
P4	65/M	2	2	A	1	1 L/0 R	13 L/0 R	33%	2	11 L/20 R	hem.	NA^b^	NA^b^
P5	67/M	3	3	A	2	6 L/0 R	4 L/1 R	54%	5	1 L/6 R	isch.	277.50	(SLF1), SLF2, SLF3, IFOF, AF
P6	69/M	2	2	A	1	14 L/2 R	11 L/2 R	26%	1	1 L/7 R	isch.	364.26	SLF2, SLF3, IFOF, ILF, AF
P7	67/M	3	3	P	2	13 L/2 R	10 L/5 R	49%	5	3 L/13 R	isch.	168.62	SLF2, SLF3, IFOF, ILF, AF

Brackets indicate partial disconnection; NA, not available;

a artifact on diffusion sequences;

b *contraindication for MRI; MD, motor deficit (0, absent; 1, monoparesis; 2, incomplete hemiparesis; 3, complete); SSD, somatosensory deficit (0, absent; 1, superficial; 2, incomplete superficial and deep; 3, complete); LHH, left homonymous hemianopia (A, absent; P, present); OCD, right ocular and cephalic deviation (0, no deviation; 1, spontaneously reducible; 2, reducible under order); LCT, Line cancelation test (Albert, 1973), XL = omission on left side/18 and XR = omission on right side/18; BCT, Bells cancelation test (Gauthier et al., 1989), XL = omission on left side/15 and XR = omission on right side/15; LBT, Line bisection task, score is the mean percentage of deviation toward the right with a positive sign or toward the left with a negative sign (Schenkenberg et al., 1980); CDT, Copy drawing task (Gainotti et al., 1972), number of omission/10; DDFM: daisy drawn from memory XL = number of petals on left side and XR = number of petals on right side; Etiology (Isch, ischemia; Hem, hemorrhage); SLF, superior longitudinal fasciculus; IFOF, inferior fronto-occipital fasciculus; ILF, inferior longitudinal fasciculus; AF, arcuate fasciculus*.

**Figure 2:**
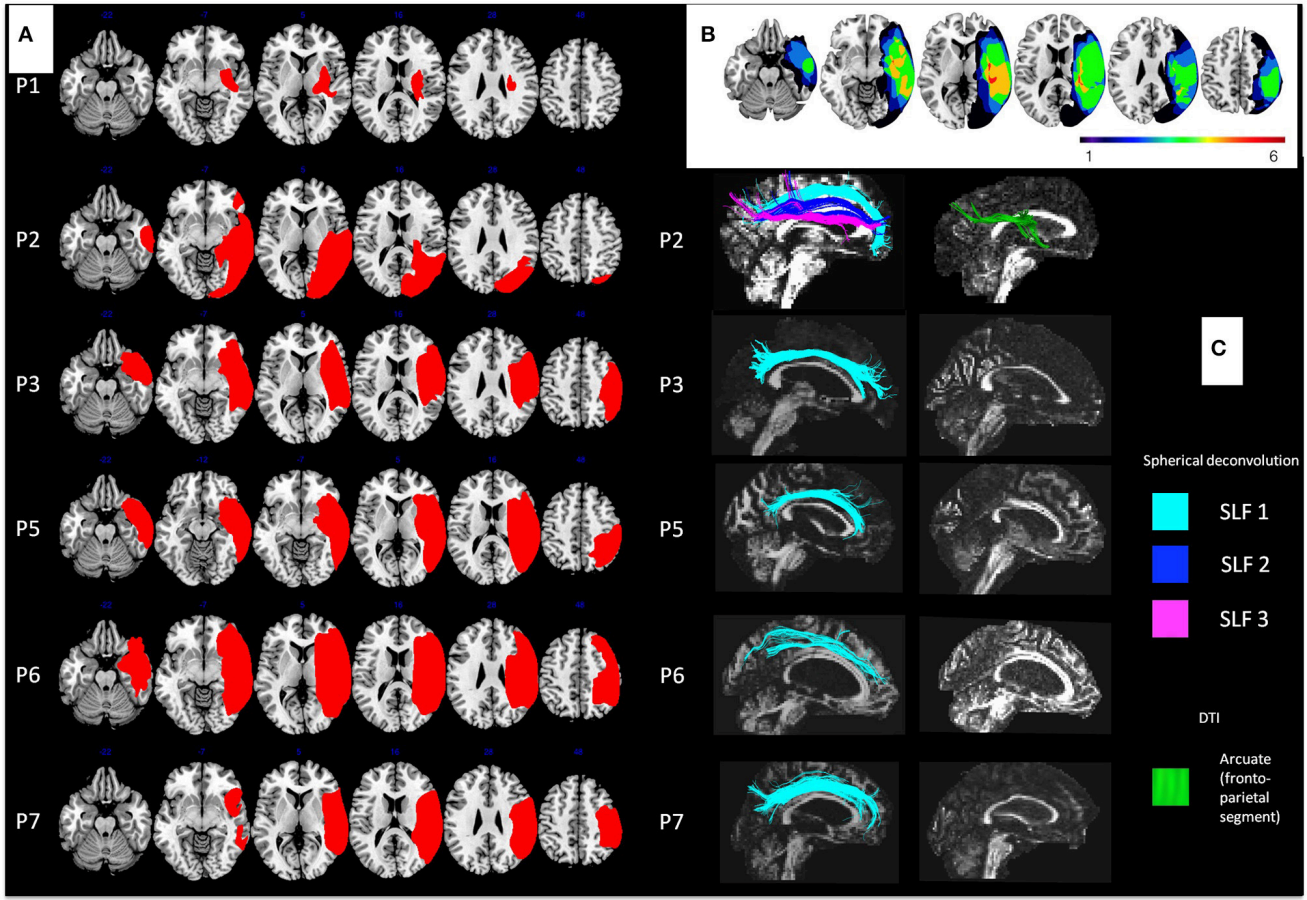
Description of lesions in patients. **(A)** Reconstruction of the brain lesions for each patient (P1–P7) in MNI space. **(B)** Overlap of the six brain lesions. The color range indicates the number of patients with lesion for each voxel. **(C)** Right intra-hemispheric networks in each patient. On the left spherical deconvolution dissection of white matter tracts shows relative integrity of the first branch of the superior longitudinal fasciculus (in light blue) in all patients, and relative integrity of the three branches in patient P2 (SL2 in dark blue and SLF 3 in pink). DTI dissection of white matter tracts on the right shows important damage of the ventral network, with only the presence of the fronto-parietal segment of the arcuate (in green) in one patient (P2). For all patients analyzed, their lesions disconnected completely the inferior fronto-occipital and the inferior longitudinal fasciculi and the fronto-temporal segment and the temporo-parietal segment of the arcuate fasciculus.

For each patient, topography of the brain lesion was studied by an anatomical MRI (excepted for one patient who presented a contraindication of MRI examination), and diffusion weighted imaging (DWI) was performed in order to objectify the different white matter tracts impaired, as previously reported (Lunven et al., 2014), and described in Supplementary Methods. As reported in Table 1 and Figure 2, most patients suffered from large lesions in the right hemisphere. The maximum overlap (5 of 6 patients) concerned the subcortical white matter, the insula and the superior temporal lobe. Damage of the parietal lobe, including the supramarginal and angular gyri, inferior frontal lobe, the inferior/middle temporal gyri and the putamen, was present in 4 of 6 patients. DWI was not available for patient 1 (P1) because of an artifact in the acquisition that we could not correct. White matter pathway reconstruction was possible in 5 of 6 patients. Four patients (P3, P5, P6, and P7) had a lesion of the whole 2nd and 3rd branches of the superior longitudinal fasciculus (SLF) (Thiebaut De Schotten, 2012). In one case (P2), the SLF was traceable but the patient had direct damage of the splenium and her neglect was described as a consequence of an inter-hemispheric disconnection (Lunven et al., 2014).

Ten right-handed healthy controls were included in this study (six men, four women; 50–72 years, mean 63.3 years, *SD* = 5.9) to assess learning effect in the VR task, and performed the same tasks (i.e., VR task and drawing from memory) at the same time points (Figure 1).

### Virtual supermarket VAP-S task

#### The VAP-S virtual software and environment

**Figure 3:**
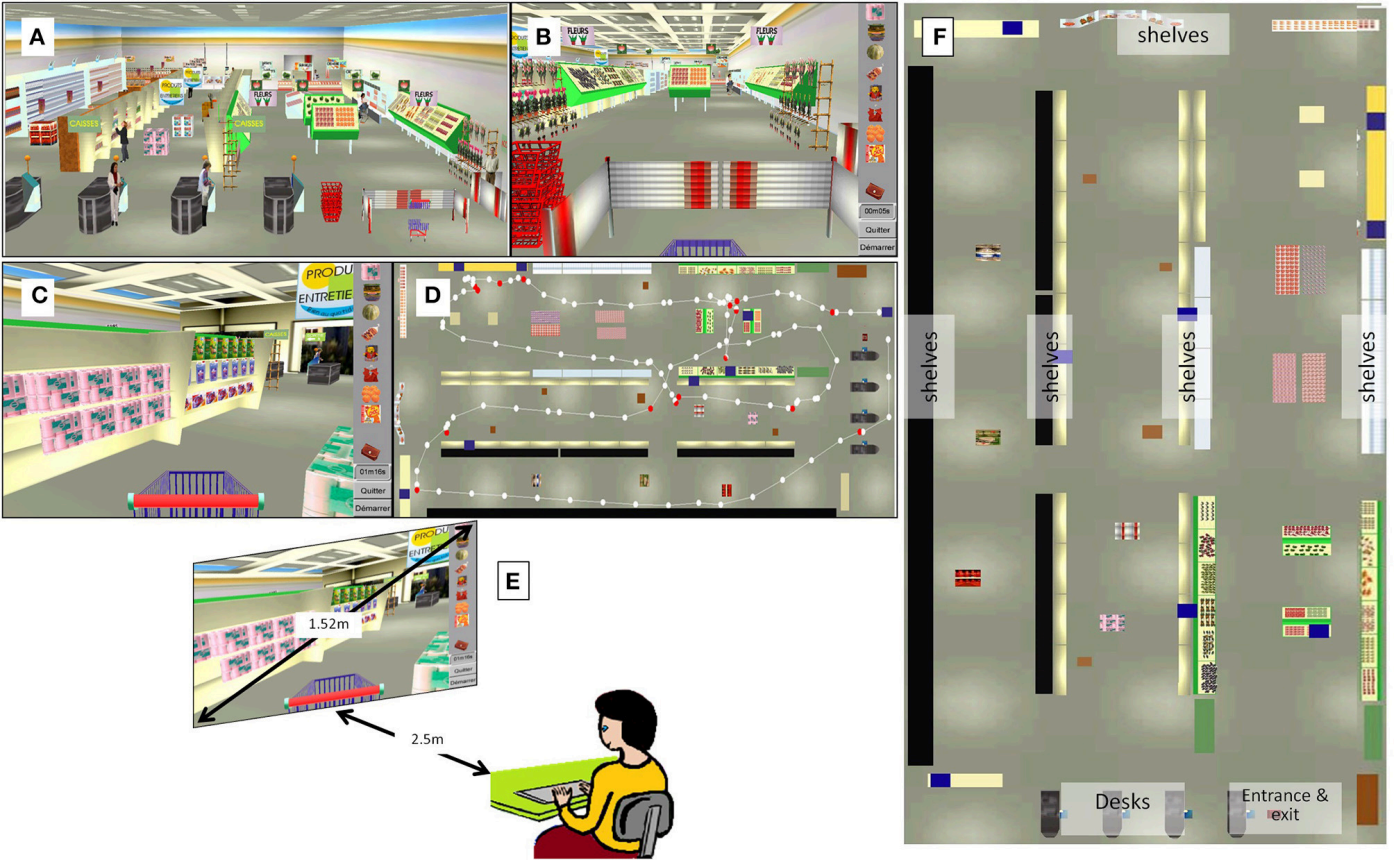
Virtual supermarket VAP-S. **(A)** Overall view of the supermarket. It simulated a textured virtual medium-sized supermarket with multiple shelves of drinks, food, cleaning materials, etc., and four checkouts, a reception point, and a cart. Some obstacles, such as packs of bottles or customers were designed to obstruct the advancement of participants in the various aisles. **(B)** View of the supermarket entrance with the list on the right side and the cart. **(C)** View of an aisle with an item to purchase. **(D)** View of the supermarket map to analyze the session, with items to purchase (blue squares), pathway, and pauses (red points). **(E)** Set-up of the procedure. **(F)** The “ideal” map of the layout of the virtual environment.

VR task was assessed in the virtual supermarket VAP-S (Klinger et al., 2006). The VAP-S simulated a textured virtual medium-sized supermarket with multiple shelves of drinks, food, cleaning materials, etc., four desks, a reception point, and a cart (Figure 3). Some obstacles, such as packs of bottles or customers were designed to obstruct the advancement of participants in various aisles. The VAP-S task is an original paradigm, similar to the “shopping list test” (Martin, 1972), during which the participant is asked to purchase items from a clearly marked list of products, then proceed to the cashier's desk, and pay for them.

The participant was sitting in a dark room and the virtual environment was projected onto a 60-inch 4/3 screen (Figure 3). He/she had to enter the supermarket, positioned in front of the cart, find the items on a shopping list, place these in the cart, and purchase these at the checkout. A standard computer keyboard was used to move within the supermarket, and items were placed in the cart by selecting them with a cursor (mouse click). The list of items was composed of eight articles presented on various shelves, four on the right side and four on the left side of the shop. It was displayed on the right side of the screen (Figure 3). The patient was regularly reminded orally by the investigator of the items yet to be purchased. A randomized new list was used for each session (i.e., the two pre-tests and the four post-tests) to avoid a learning effect. When the participant selected the item, it disappeared from the displayed list and appeared in the cart. For each pre or post-test, the participant had to buy items as quickly as possible, to pay, and to leave the store. Each test lasted a maximum of 45 min owing to the fatigability of the patients. Before the first pre-test, each participant had a period of familiarization of 2 days with the virtual tool, with several VR training sessions and various lists (from 1 to 8 items), to improve his/her performance in the virtual environment. These training tasks were performed with the assistance of the investigator and written instructions given by the software. This assistance decreased gradually, in order to reach a plateau of performance.

### Outcome measures of the VAP-S

For each session, six parameters reflecting the navigation in the virtual supermarket were measured: (i) the total distance (meters), (ii) the total duration of session (minutes), (iii) the number of omissions (i.e., each time the patient walked past an item and neglected it), (iv) the total number of items purchased, (v) the number of items purchased on the right and on the left, considering the trajectory of the participant, and (vi) the number of pauses. The position of the participant was recorded by the VAP-S, which provided the display of his/her pathway on a map of the supermarket, and allowed an automated measurement of parameters.

### Drawing from memory

In order to assess generalization of PA effects to the virtual spatial domain and representation of space and topographic memory, each participant was asked to draw from memory the map of the VAP-S on an A3-sized sheet of paper. Instructions were given orally, and the participant had to draw freely his/her own representation of the map, mentally created after the navigation task, reflecting the ability to create an allocentric representation of space. The participant was asked to draw the map, place landmarks if possible (i.e., the entrance, the exit, the aisles, and desks, etc.,), and any item, bought or not, that he/she could localize on the map. No item or landmarks were reminded by the investigator. Figure 3F provides the map of the virtual environment.

Five parameters were measured: (i) lateral index (LI) that measures the length of the lines drawn on the right and left part of the sheet (LI = $\frac{right\text{ lines drawn}-left\text{ lines drawn}}{right\text{ lines drawn}+left\text{ lines drawn}}$; deviation toward the right is indicated by a positive value or toward the left by a negative value), considering the symmetry axis of the sheet in reference to a egocentric frame, and (ii) LI that measures the length of the lines drawn on the right and left part of the drawing map, considering the symmetry axis of the drawing map in reference to a allocentric frame, (iii) the number of purchased and non-purchased items drawn on the map, (iv) the number of items drawn on the left and right side of the map considering it's symmetry axis, and (v) the number of items correctly located on the map. No feedback of the performance was given in order to avoid any bias due to motivation.

### Prism adaptation

#### Adaptation procedure

Patients were exposed to a rightward optical shift of the visual field produced by the prismatic lenses. Glacier goggles (Julbo®, Lyon, France) were fitted with wide-field, point-to-point wedge lenses creating an optical shift of 10° (http://OptiquePeter.com, Lyon, France) affording wide binocular vision. The total visual field was 110° and the one-eye visual field was 80° (including a 50° binocular field). With these goggles on, the visual field was uniformly displaced to the right with minimal visual distortion. The exposure period consisted of pointing responses to visual targets presented 10° to the right or to the left of the objective body midline. During the prism exposure, each patient was asked to point at a fast but comfortable speed; he/she could see the target, the second half of the pointing trajectory and the terminal error. His/her head was kept aligned with the sagittal axis of the body by a chin-rest and controlled by an investigator throughout the adaptation procedure. The total duration of this exposure was 15–20 min (at least 200 pointing movements per session), and each patient had 10 sessions over 2 weeks (for details see Rode et al., 2015).

### Subjective straight-ahead (SSA) pointing test

SSA was evaluated by a simple manual pointing task performed without visual feedback to assess proprioceptive adaptation. The blindfolded patient was seated in front of a horizontal box in the darkness that permitted measurements of the finger movement endpoints with an accuracy of 1°. Patients were required to point straight-ahead with his/her right arm while his/her head was kept aligned with the sagittal axis of the body. The mean of at least 10 pointing was measured at each test (for details see Rossetti et al., 1998).

### Analyses

Analysis of variance was used to compare performance between the two pre-tests to ensure that the period of familiarization permitted to reach a plateau. Then, in order to see whether the performances of patients reached those of controls we compared them using independent *T*-tests.

First, in order to assess the effects of PA on VR and memory tasks, we compared performance before and after PA using a repeated-measures analysis of variance (one way ANOVA) taking into account variability between sessions. Then, in order to distinguish in more detail between short-term and long-lasting improvement after PA, we conducted planned comparison between pre-tests and each post-test. We also performed the same analyses among controls, in order to explore a possible learning effect. We explored PA after-effect analyzing SSA measures with a same statistical procedure. Finally, we performed same analyses for paper-and-pencil tests, including tests at the inclusion as a pre-test. Statistical significance was set at *p* < 0.05. Statistical analyses were performed with the software STATISTICA® (StatSoft France, 2008).

## Results

### Subjective straight-ahead and neglect pen-and-pencil tests

In pre-tests, the mean (*SD*) SSA of the patients was 9.89° (1.64), reflecting a shift of egocentric reference toward the right side. No significant difference was found between the two pre-tests [*F*_(2, 17)_ = 0.048, *p* = 0.953]. In post-tests, the mean (*SD*) values were 2.12° (1.76) at 0 h post-test, 5.99° (1.95) at day+3, 5.82° (1.35) at day+7, and 4.59° (1.83) at day+30. The mean value of this shift was significantly reduced after therapy [η^2^ = 51.1%; *F*_(6, 24)_ = 4.18; *p* = 0.005]; the greatest difference was found at post-test 0 h [η^2^ = 81.2%; *F*_(1, 4)_ = 17.24; *p* = 0.014], indicating the proprioceptive adaptation after PA. This reduction remained significant at post-test day+30 [η^2^ = 75.6%; *F*_(1, 4)_ = 12.40; *p* = 0.024].

In pre-tests, all patients showed a left visual neglect assessed by the paper-and-pencil tasks (see Table 1). In post-tests, a significant decrease of the total number of omissions was noted for the line cancelation test [η^2^ = 28.6%; *F*_(6, 36)_ = 2.403; *p* = 0.047]. Moreover, a significant improvement of the percentage deviation toward the right was demonstrated for the bisection task [η^2^ = 42.1%; *F*_(6, 36)_ = 4.37; *p* = 0.002]. The improvement in the bisection task was maintained at post-test day+3 [η^2^ = 55.2%; *F*_(1, 6)_ = 7.40; *p* = 0.035] and post-test day+7 [η^2^ = 74%; *F*_(1, 6)_ = 17.12; *p* = 0.006], and the improvement in the line cancelation test was maintained at day+30 regarding the total number of omissions [η^2^ = 56.2%; *F*_(1, 6)_ = 7.683; *p* = 0.032].

**Figure 4:**
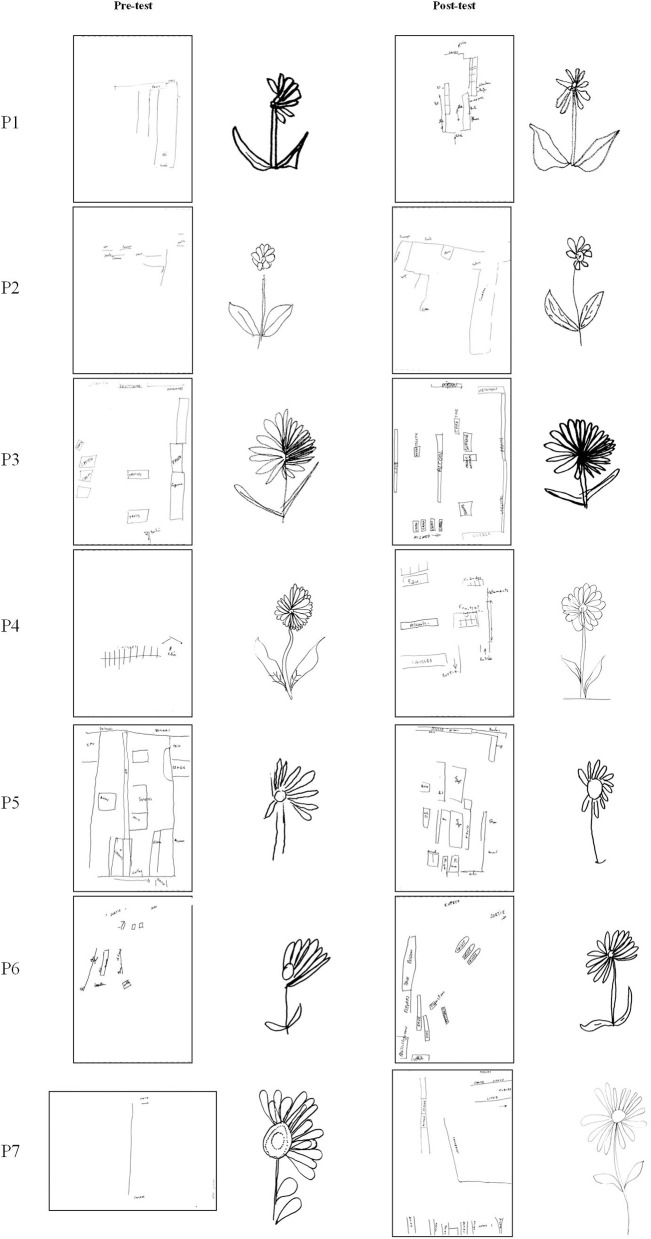
Drawings of daisy and virtual supermarket map from memory. Example of drawings made by each neglect patient before and after prism adaptation.

For the drawing a daisy from memory task, patients drew more petals on the left side of the daisy [η^2^ = 38.1%; *F*_(6, 36)_ = 3.701; *p* = 0.006], but not on the right side [η^2^ = 9.7%; *F*_(6, 36)_ = 0.644; *p* = 0.695]. The qualitative analysis of drawings shows a reduction of the left-sided object centered neglect in 3 patients (P1, P6, and P7) and an enlargement of the left half of the daisy (misrepresentation) in one case (P4), reflecting an effect of PA on representational deficit. This improvement was maintained at day+30 [η^2^ = 81.9%; *F*_(1, 6)_ = 25.214; *p* = 0.002; Table 2]. Examples of daisy drawn are presented in Figure 4.

**Table 2:** Virtual supermarket task and drawing map for the seven patients. Data presented are mean and between brackets standard deviation.

	**PRE-TESTS**		**POST-TESTS**			
	**Day−4**	**Day−2**	**0 h**	**Day+3**	**Day+7**	**Day+30**
**PAPER-AND-PENCIL TESTS**						
Line cancelation test	11.43 (5.53)	8.86 (6.41)	5.86 (2.54)^†^	7.86 (3.89)^†^	9.43 (7.68)	5.29 (1.98)^†^
Bells cancelation test	17.43 (3.31)	22 (4.51)	19.86 (4.26)	21.29 (4.07)	21.71 (4.75)	22.29 (4.72)
Line bisection task	22% (0.29)	10% (0.25)	10% (0.24)	8% (0.22)^†^	7% (0.23)^‡^	16% (0.25)
Copy drawing task	3.86 (2.61)	3.71 (3.09)	2.71 (2.98)	2 (2.65)	2.43 (2.76)	3.71 (3.25)
Daisy Drawn From Memory	3.43 (3.87) L/8.14 (6.31) R	5 (3.32) L/9.57 (7.46) R	6.57 (5.91) L/10.57 (6.32) R^‡^	8 (6) L/10.14 (6.52) R^‡^	5.86 (3.53) L/8.43 (3.21) R^†^	7.14 (4.95) L/9.29 (4.15) R^‡^
**VIRTUAL REALITY**						
Duration: minutes	34.45 (15.47)	32.28 (17.41)	25.97 (13.99)^‡^	27.67 (14.82)	31.16 (15.86)	25.89 (16.41)^†^
Distance: meters	553 (326)	531 (507)	376 (247)	415 (231)	433 (196)	381 (245)
Number of pauses	80 (46)	79 (51)	64 (37)^†^	66 (43)	81 (49)	61 (41)
Omissions	6.13 (4.16)	7.88 (9.39)	2.63 (1.69)	3.75 (2.49)	4.00 (3.82)	2.25 (2.05)
Items purchased	4.75 (2.76)	4.38 (2.77)	^†^6.38 (2.83)	^†^6.88 (2.80)	6.50 (2.83)^†^	6.63 (2.77)^†^
Items purchased on the left side	1.43 (1.13)	1.29 (1.11)	2.86 (1.57)	3.71 (1.8)^†^	3 (2)	2.57 (1.72)
**DRAWING MAPS FROM MEMORY**						
LI axis of sheet	0.10 (0.52)	0.32 (0.74)	0.09 (0.39)	−0.19 (0.40)	0.26 (0.54)	0.29 (0.61)
LI axis of map	0.07 (0.29)	0.39 (0.49)	−0.06 (0.22)^‡^	−0.15 (0.34)	0.17 (0.27)	0.17 (0.48)
Items drawn	6.25 (5.04)	5.86 (4.81)	9.75 (5.18)^†^	10.57 (6.68)^†^	10.50 (6.68)^†^	9.50 (7.27)
Items drawn on the left side	3.14 (2.48)	3.4 (2.07)	6.43 (3.05)^†^	6.83 (2.32)^‡^	6.43 (3.95)	7 (2.9)
Items correctly located	2.50 (3.46)	2.00 (3.61)	5.133 (0.72)^†^	4.71 (4.19)^†^	3.63 (2.45)	4.13 (4.09)

† *p < 0.05*,

‡ *p < 0.01: analysis of variance (one way ANOVA) between the pre- tests vs. the specific post-test. LI, lateral index (positive values = deviation toward the right, negative values = deviation toward the left). Line cancelation test (Albert, 1973): total number of omissions /40; Bells cancelation test (Gauthier et al., 1989), total number of omissions/35; Line bisection task, score is the mean percentage of deviation toward the right with a positive sign or toward the left with a negative sign (Schenkenberg et al., 1980); Copy drawing task (Gainotti et al., 1972), number of omissions/10; DDFM, daisy drawn from memory XL = mean number of petals on left side and XR = mean number of petals on right side*.

On the other hand, no significant improvement was evidenced for the copy drawing test [η^2^ = 22.5%; *F*_(6, 36)_ = 1.739; *p* = 0.140], and nor for the bells cancelation test despite a trend toward improvement [η^2^ = 27.3%; *F*_(6, 36)_ = 2.259; *p* = 0.059].

### Effects of prism adaptation on navigation performance in the virtual environment

No significant difference in performance between the two pre-tests was noted for all participants confirming that familiarization allowed patients to reach a performance plateau (see Supplementary Results).

Before PA, only two patients (P2 and P3) were able to finish the VR task, purchasing all the items within 45 min. After PA, six patients finished the task (P2, P3, P4, P5, P6, and P7). Regarding the overall effect of PA (i.e., pre-tests versus all post-tests), the navigation performance of patients was significantly improved after PA: the duration of session was significantly decreased [η^2^ = 37.3%; *F*_(5, 30)_ = 3.57; *p* = 0.012] as was the number of pauses [η^2^ = 33.6%; *F*_(5, 30)_ = 3.04; *p* = 0.025] and the number of omissions [η^2^ = 32.3%; *F*_(5, 30)_ = 2.87; *p* = 0.031]. No significant difference was found for the total distance [η^2^ = 0.4%; *F*_(5, 30)_ = 1.54; *p* = 0.21]. The number of items purchased was significantly increased [η^2^ = 58.2%; *F*_(5, 30)_ = 8.36; *p* < 0.001], as was the number of items purchased on the left [η^2^ = 44.3%; *F*_(5, 30)_ = 4.780; *p* = 0.002]. On the other hand, the number of items purchased on the right was not significantly increased after PA [η^2^ = 11.1%; *F*_(5, 30)_ = 0.666; *p* = 0.652].

A short-term effect of PA was found at post-test 0 h. There was a significant improvement in navigation: the duration of session was significantly decreased [η^2^ = 69.9%; *F*_(1, 6)_ = 13.9; *p* = 0.009] as was the number of pauses [η^2^ = 54.3%; *F*_(1, 6)_ = 7.13; *p* = 0.037]. The number of items purchased was significantly greater [η^2^ = 58.6%; *F*_(1, 6)_ = 8.50; *p* = 0.027].

The number of items purchased on the left was also significantly greater at post-test day+3 [η^2^ = 65.3%; *F*_(1, 6)_ = 11.294; *p* = 0.015].

A long-lasting effect of PA was found at post-test day+30; there was a significant improvement in navigation. Time to accomplish the task was significantly decreased [η^2^ = 53.3%; *F*_(1, 6)_ = 6.84; *p* = 0.039], and the number of items purchased was significantly greater [η^2^ = 65.6%; *F*_(1, 6)_ = 11.46; *p* = 0.015; Table 2].

Performance of patients was significantly lower than controls for all parameters in pre-tests. After PA, the number of items purchased by patients reached the number purchased by controls [post-test 0 h: *t*_(15)_ = 1.828, *p* = 0.087; post-test day+3: *t*_(15)_ = 1.213, *p* = 0.244; post-test day+7: *t*_(15)_ = 1.617, *p* = 0.127; post-test day+30: *t*_(15)_ = 1.748, *p* = 0.101]. Table 3 summarizes results for control subjects. Among these, there was a lower performance for the navigation task at post-test 0 h as evidenced by a greater distance covered to find items [η^2^ = 34.2%; *F*_(5, 35)_ = 3.633; *p* = 0.009], the greater duration of session [η^2^ = 32.6%; *F*_(5, 35)_ = 3.389; *p* = 0.013], the greater number of pauses [η^2^ = 31.3%; *F*_(5, 35)_ = 3.188; *p* = 0.018], thus indicating the absence of a learning effect between sessions.

**Table 3:** Means of parameters of the virtual supermarket task and drawing maps for control group. Data presented are mean and between brackets standard deviation.

	**PRE-TESTS**		**POST-TESTS**			
	**Day−4**	**Day−2**	**0 h**	**Day+3**	**Day+7**	**Day+30**
**VIRTUAL REALITY**						
Duration: minutes	6.61 (1.46)	8.46 7 (2. 2)	10 (4.88)	7.8 (2.45)	7.4 (1.4)	6.85 (1.68)
Distance: meters	149.96 (6.83)	194.75 (58.97)	242.72 (86.76)^†^	190.57 (59.38)	161.12 (32.44)	162.65 (40.21)
Number of pauses	17 (4.42)	20.30 (7.13)	23.80 (13.45)	19 (4.78)	16.25 (3.06)	16.44 (4)
Omissions	0 (0)	0.50 (0.53)	0.70 (0.95)	0.50 (0.71)	0.10 (0.32)	0.60 (0.70)
Item purchased	8 (0)	8 (0)	8 (0)	8 (0)	8 (0)	8 (0)
LI of item purchased	0.95 (0.32)	1.12 (0.68)	1.01 (0.39)	1.29 (0.68)	0.55 (0.29)	1.1 (0.34)
**DRAWING MAPS FROM MEMORY**						
Items drawn	34.10 (7.13)	33.60 (5.85)	29.30 (5.91)^†^	29.70 (5.42)	35.00 (5.57)	32.40 (7.52)
Items correctly located	30.10 (6.37)	30.20 (7.38)	25.60 (5.40)	27 (5.79)	30.89 (7.25)	29.2 (8.12)

† *p < 0.05, analysis of variance (one way ANOVA) between the pre- tests VS the specific post-test. LI, lateral index*.

### Effects of prism adaptation on representation of space and topographic memory

Before PA, the patients had difficulties drawing from memory the map of the virtual supermarket. Drawings revealed a right shift of the symmetry axis of the map (allocentric frame), a right shift regarding the axis of the A3 sheet (egocentric frame) and constructional apraxia (Table 2 and Figure 4). Regarding the overall effect of PA (i.e., pre-tests versus all post-tests), the representation of space and topographic memory of patients were improved after PA: the right shift of the symmetry axis of the map was significantly reduced [η^2^ = 36.1%; *F*_(5, 25)_ = 2.83; *p* = 0.037]. Moreover, significant effects of PA were found for the total number of items drawn [η^2^ = 46.0%; *F*_(5, 25)_ = 4.26; *p* < 0.001], the number of items drawn that were previously purchased in the VR task [η^2^ = 36.5%; *F*_(5, 25)_ = 2.868; *p* = 0.035], the number of items drawn that were not identified in the VR task [η^2^ = 41.2%; *F*_(5, 25)_ = 3.502; *p* = 0.016], the number of items drawn on the left [η^2^ = 56.2%; *F*_(5, 25)_ = 6.404; *p* = 0.001], and the number of items correctly located on the map [η^2^ = 41.8%; *F*_(5, 25)_ = 3.59; *p* = 0.013]. No significant difference in the axis of the A3 sheet was found after PA [η^2^ = 25.8%; *F*_(5, 25)_ = 1.74; *p* = 0.16] and for the number of items drawn on the right [η^2^ = 11.7%; *F*_(5, 25)_ = 0.663; *p* = 0.655].

A short-term effect of PA was noted at post-test 0 h; there was an improvement of the representation of space and topographic memory: the right shift of the symmetry axis of the map was significantly reduced [η^2^ = 91.3%; *F*_(1, 5)_ = 11.71; *p* < 0.001], and a significant increase was found in the total number of items drawn [η^2^ = 59.5%; *F*_(1, 5)_ = 7.34; *p* = 0.042], as well as the number of items drawn on the left [η^2^ = 71.1%; *F*_(1, 5)_ = 12.273; *p* = 0.017] and the number of items correctly located on the map [η^2^ = 69.7%; *F*_(1, 5)_ = 11.48; *p* = 0.019].

The number of items drawn on the left was also significantly greater at post-test day+3 [η^2^ = 84.7%; *F*_(1, 5)_ = 27.769; *p* = 0.003].

A long-lasting effect of PA was found at post-test day+7; there was an improvement of topographic memory, as demonstrated by a significant increase in the total number of items drawn [η^2^ = 69.4%; *F*_(1, 5)_ = 11.32; *p* = 0.02]. A long-lasting effect of PA was also suggested at post-test day+30; there was a trend toward improvement in the representation of space and topographic memory after PA: a trend toward an increase was found in the total number of items drawn [η^2^ = 53.3%; *F*_(1, 5)_ = 5.70; *p* = 0.062] as well as the number of items correctly located on the map [η^2^ = 56.8%; *F*_(1, 5)_ = 6.58; *p* = 0.051; Table 2]. Examples of maps drawn are presented in Figure 4. All the drawings of the map are presented in Supplemental Results (Supplementary Figure 1).

Performance in the topographic memory task was significantly lower in post-tests after 2 weeks without using the VR tool for control subjects (Table 3), as evidenced by the significantly fewer number of items drawn on the map at post-test day 0 h [η^2^ = 32%; *F*_(5, 40)_ = 3.763; *p* = 0.007] as well as the number of items correctly located on the map [η^2^ = 28.9%; *F*_(5, 40)_ = 3.244; *p* = 0.015].

## Discussion

The aim of this study was to assess the effects of PA on navigation in a virtual environment and on the representation of space and topographic memory of right-brain damaged patients with chronic neglect. The results indicate that PA reduces the rightward attentional bias in a VR task and improves navigation and topographic memory, and that these improvements persist over time.

Before PA, neglect patients showed a behavioral bias with poor ability to find and select items located on the left side in the virtual supermarket. Navigation in the supermarket was a difficult task for all patients, and only two of them could achieve the task within the 45 min time limit, whereas control subjects did so in less than 10 min. After PA, navigation was improved, and all patients but one were able to finish the task, and they did so more rapidly, with fewer pauses. The improvement was also demonstrated by the greater number of items purchased, the greater number of items located on the neglected side, and fewer omissions. Hence, this lateralized effect of PA on the selection of items located in the neglect side brings direct evidence that prisms may have led to a leftward reorientation of attention in the patient group. This could be explained by a reduction of the rightward attention bias after PA; it is unlikely that this was due to a training effect as familiarization allowed participant performance to reach a plateau, purchased items on the right were not increased and results of control subjects suggest that aspects regarding the supermarket were forgotten over the 2 weeks without use of the VR tool. However, although these findings suggest that this improvement could be related to reduction of rightward attention bias, a direct effect of PA on navigation abilities still remains unclear as highlighted by the non-significant change in the distance covered to find items. Finally, it may be that PA also improved attentional resources such as sustained attention and/or arousal (Ricci et al., 2016). However, the lack of significant increase of purchased items on the right in the VR task is not in favor of this hypothesis.

The improvement of navigation and topographic memory could be explained by the reduction of the rightward attentional bias. Before PA, patients had difficulties drawing the map of the virtual supermarket from memory. Drawings revealed a neglect of the left part of the sheet, omissions of left-sided items and constructional apraxia consistent with a left representational deficit. After PA, the drawings and their graphic characteristics, such as symmetry or line orientation were improved, more items were drawn and items were better located, and the neglected part of sheet was reduced. These positive effects were reported although this task was not included in the VR task. This suggests an expansion of effects of PA to high-level of supramodal representations of space and visuoconstructive abilities associated with reduction of the rightward attentional bias (Rode et al., 2006).

The patients were also able to place more items on their map immediately and 1 week after PA, remembering more information from long-term memory in addition to be able to draw a more symmetrical space. As for improvement in navigation, these results cannot be explained by a learning effect. Indeed, this improvement was noted for items drawn on the left side of the map and not on the right side, indicating a reduction of the rightward bias of spatial representation and topographic memory after PA. Before PA, patients had a representational deficit and poor ability to mentally explore the left side of space, but also to access semantic information in connection with this part of space (Rode et al., 2004, 2010). PA increases topographic information and memory, but also the location of this information suggesting an improvement of these two aspects of representation: topographic and semantic. We can suppose that the re-orientation of the attentional bias toward the left side after PA favored the building of more symmetrical spatial representation of map and the access to topographic information in relation to the part of this space, as if the positive effects of PA on processes involved in spatial localization (“where”) facilitate the recall of semantic knowledge (“what”). This facilitation might also involve of the recall from implicit memory, as suggested by the greater number of items drawn that were not purchased in the VR task (Bisiach et al., 1999). After-effects of PA could therefore improve abilities to create a representation of space from an egocentric exploration of virtual space, as well as its manipulation and topographic memory. Neglect patients are known to fail to generate and to use a mental representation of space (Bisiach and Luzzatti, 1978; Rode et al., 2010) and to perform mental transformations of an egocentric versus allocentric spatial representation, and *vice versa* (Palermo et al., 2012).

Several limitations should be acknowledged. Our observations reflect results derived from a small group of patients. Moreover, these findings were not compared to a control group of neglect patients. However, in order to counterbalance this lack of control group, the design of the study included 2 pre-tests performed 2 days apart after a long period of familiarization in order to reach a plateau of performance, confirmed by the absence of difference between the two pre-tests, and to be sure that the improvement could not be explained by learning the task and improving over time.

Nevertheless, the results show an improvement persisting up to 1 month after PA. These long-lasting effects were obtained in a task that does not involve visuo-manual ability, reinforcing the hypothesis that after-effects of PA could expand to a high level of space representation by a bottom-up track. They also reinforce the idea that a few repeated PA sessions are more effective than a single session (Frassinetti et al., 2002; Humphreys et al., 2006; Shiraishi et al., 2008; Serino et al., 2009). Furthermore, herein the improvement was reported in the virtual environment, underlying the potential interest of this kind of approach for cognitive rehabilitation of neglect. Further studies are needed to confirm these preliminary findings.

## Disclosure

Preliminary results were presented at the 5th International Conference on Spatial Cognition “Space and Embodied Cognition.” Roma, September 4–8, 2012.

## Ethics statement

This study was carried out in accordance with the recommendations of “Comités de Protection des Personnes Sud-Est” with written informed consent from all subjects. All subjects gave written informed consent in accordance with the Declaration of Helsinki. The protocol was approved by the “Comités de Protection des Personnes Sud-Est.”

## Author contributions

BG and GR: designed the work acquired, analyzed, did interpretation of data for the work, drafted the work; ML, YR and SJ-C: did interpretation of data for the work, revised the work critically for important intellectual content; PR: analyzed data for the work, revised the work critically for important intellectual content; EK and P-AJ: designed the work, revised the work critically for important intellectual content.

All authors approved the final version to be published, and agree to be accountable for all aspects of the work in ensuring that questions related to the accuracy or integrity of any part of the work are appropriately investigated and resolved.

### Conflict of interest statement

The authors declare that the research was conducted in the absence of any commercial or financial relationships that could be construed as a potential conflict of interest. The reviewer, RR, and handling Editor declared their shared affiliation.

## Supplementary material

Supplementary Figure 1 Drawings of virtual supermarket map from memory. Drawings made by each neglect patient before (pre-tests day −4 and day −2) and after (post-tests immediate, day +3, day +7, and day +30) prism adaptation.
