# Supplementary figures and images for: Improvement of Navigation and Representation in Virtual Reality after Prism Adaptation in Neglect Patients
Source: Front Psychol. 2017 Nov 20;8:2019. doi: 10.3389/fpsyg.2017.02019 (PMC5701812; doi:10.3389/fpsyg.2017.02019)

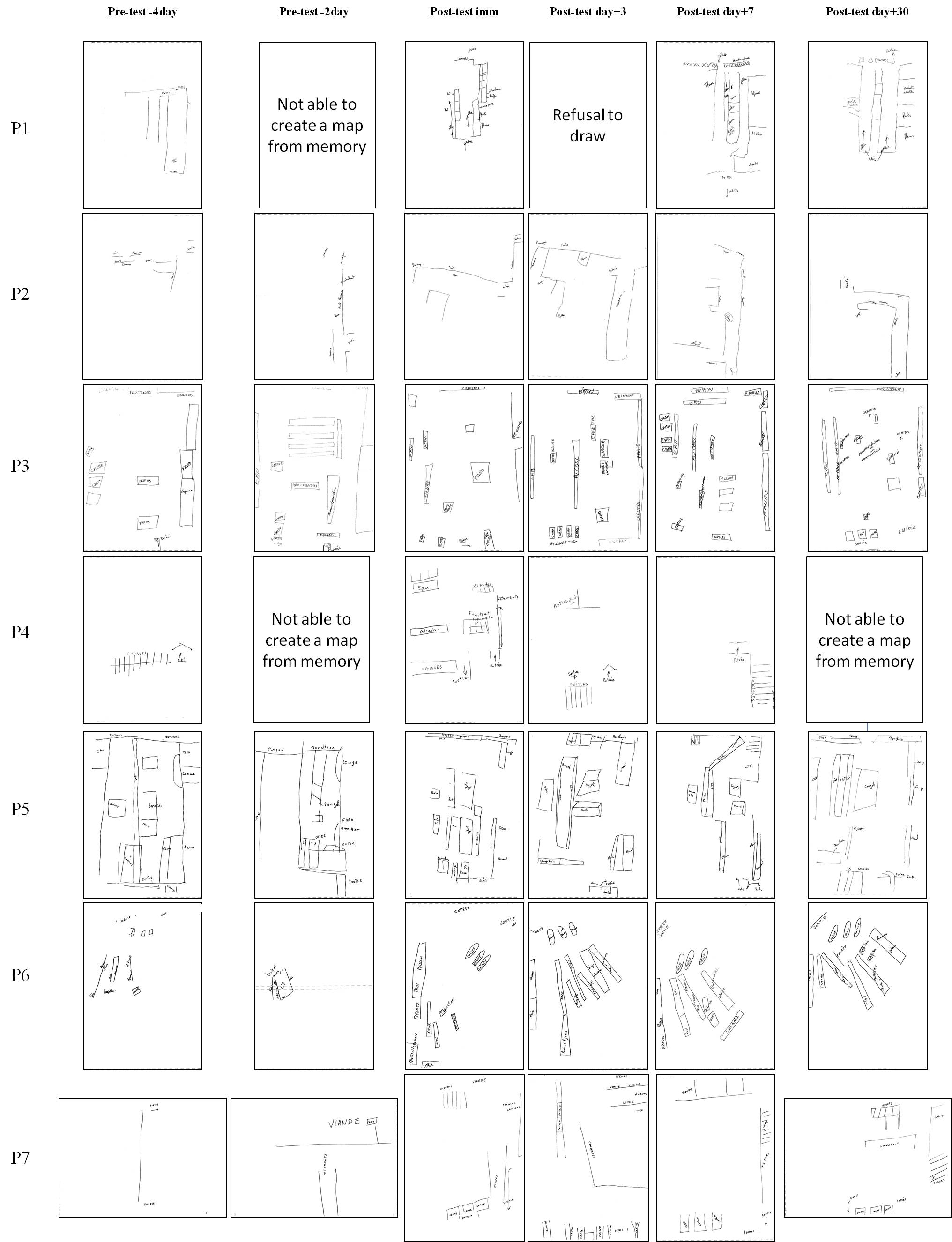

Supplement: Supplementary Figure 1 — Drawings of virtual supermarket map from memory. Drawings made by each neglect patient before (pre-tests day −4 and day −2) and after (post-tests immediate, day +3, day +7, and day +30) prism adaptation. [file Image1.jpeg]
