# Supplementary material for: Improvement of Navigation and Representation in Virtual Reality after Prism Adaptation in Neglect Patients
Source: Front Psychol. 2017 Nov 20;8:2019. doi: 10.3389/fpsyg.2017.02019 (PMC5701812; doi:10.3389/fpsyg.2017.02019)
Supplement: Supplementary file 2 [file DataSheet1.docx]

**Supplementary methods**

1. **SUPPLEMENTARY METHODS**
   1. **MRI data acquisition:**

A sequence of Diffusion Weighted Imaging (DWI) with 60 directions with a b-value of 1500 sec mm^−2^ and one volume with no diffusion gradient were acquired on a 3T Ingenia Philips medical system equipped with a 16 channel head coil (Philips Medical Systems, Erlangen, The Netherlands). The acquisition sequence was fully optimized for tractography, providing isotropic resolution (2 × 2 × 2 mm) and coverage of the whole brain. Additionally, a three-dimensional T1 weighted covering the whole head was also acquired (187 slices, voxel resolution = 0.85 × 0.75 × 0.75 mm, TE = 4.5, TR = 9.7).

- 1. **Study of grey matter**

A mask of patient’s lesion were drawn on the native 3D T1 images using the MRIcro software (Rorden and Brett, 2000) and a graphic tablet (WACOM Intuos A6, Vancouver, Washington, USA). T1 images were normalized to a standard brain template (Montreal Neurological Institute, <http://www.mni.mcgill.ca/>) using rigid and elastic deformation tools provided in the software package Statistical Parametric Mapping 8 (SPM8, <http://www.fil.ion.ucl.ac.uk/spm>). Deformation were applied to the whole brain except for the voxels contained in the lesion mask in order to avoid deformation of the lesioned tissue (Brett et al., 2001; Volle et al., 2008). Finally, lesions of patients were drawn on the normative 3D T1.

- 1. **Study of white matter**

In the first step, we corrected simultaneously diffusion datasets for motion and geometrical distorsions using ExploreDTI (<http://www.exploredti.com>) (Leemans and Jones, 2009). The tensor model was fitted to the data using the Levenberg-Marquardt non-linear regression (Marquardt, 1963).

Whole-brain tractography was performed using an interpolated streamline algorithm (Catani et al. 2002) that propagates from voxel to voxel following a step lengh of 0.5 mm and an angle threshold less than 35°. We excluded from the tractography voxel showing an FA value inferior to 0.2 (Jones, 2004). The whole brain tractography was imported to “TrackVis” software (<http://www.trackvis.org>) (Wedeen et al., 2008) using a home-made software written in Matlab 2009b (<http://www.matworks.com>). ROIs were manually drawn on cerebral regions considered like obligatory ways for tracts of interest (Catani and Thiebaut de Schotten, 2008). The following tracts were reconstructed: the arcuate fasciculus (AF) the inferior fronto-occipital fasciculus (IFOF), and the inferior longitudinal fasciculus (ILF).

Concerning the tractography of the three branches of the SLF, we performed spherical deconvolution using a modified (damped) version of the Richardson-Lucy algorithm (Dell’Acqua et al., 2010) implemented in StarTrack software (<http://www.natbrainlab.com>) with parameters previously reported in Thiebaut de Schotten et al. (2012). A multiple-ROIs approach was used for the tractography of each branch of the SLF (for details, see Thiebaut de Schotten et al., 2011).

1. **SUPPLEMENTARY RESULTS**

The performances between the two pre-tests were not statistically different for the following data investigating navigation and representation and topographic memory: Distance (F(1, 12)=0.014, p=0.909), Duration   (F(1, 12)=0.207, p=0.657), Items purchased (F(1, 12)=0.129, p=0.726), Number of stops (F(1, 12)=0.007, p=0.937), Omissions (F(1, 12)=0.269, p=0.613), LI of items purchased (F(1, 12)=2.7, p=0.126), LI axis of sheet (F(1, 11)=0.208, p=0.657), LI axis of map (F(1, 11)=0.589, p=0.459), Items drawn (F(1, 11)=0.015, p=0.906), Items correctly located (F(1, 11)=0.065, p=0.804).

**References**

Brett M, Leff AP, Rorden C, Ashburner J. Spatial normalization of brain images with focal lesions using cost function masking. NeuroImage 2001; 14: 486–500.

Catani M, Thiebaut de Schotten M. A diffusion tensor imaging tractography atlas for virtual in vivo dissections. Cortex J. Devoted Study Nerv. Syst. Behav. 2008; 44: 1105–1132.

Dell’acqua F, Scifo P, Rizzo G, Catani M, Simmons A, Scotti G, et al. A modified damped Richardson-Lucy algorithm to reduce isotropic background effects in spherical deconvolution. NeuroImage 2010; 49: 1446–1458.

Jones DK. The effect of gradient sampling schemes on measures derived from diffusion tensor MRI: a Monte Carlo study. Magn. Reson. Med. Off. J. Soc. Magn. Reson. Med. Soc. Magn. Reson. Med. 2004; 51: 807–815.

Leemans A, Jones DK. The B-matrix must be rotated when correcting for subject motion in DTI data. Magn. Reson. Med. Off. J. Soc. Magn. Reson. Med. Soc. Magn. Reson. Med. 2009; 61: 1336–1349.

Marquardt DW. An Algorithm for Least-Squares Estimation of Nonlinear Parameters. J. Soc. Ind. Appl. Math. 1963; 11: 431–441.

Rorden C, Brett M. Stereotaxic display of brain lesions. Behav. Neurol. 2000; 12: 191–200.

Thiebaut de Schotten M, Dell’Acqua F, Forkel SJ, Simmons A, Vergani F, Murphy DGM, et al. A lateralized brain network for visuospatial attention. Nat. Neurosci. 2011; 14: 1245–1246.

Volle E, Kinkingnéhun S, Pochon J-B, Mondon K, Thiebaut de Schotten M, Seassau M, et al. The functional architecture of the left posterior and lateral prefrontal cortex in humans. Cereb. Cortex N. Y. N 1991 2008; 18: 2460–2469.

Wedeen VJ, Wang RP, Schmahmann JD, Benner T, Tseng WYI, Dai G, et al. Diffusion spectrum magnetic resonance imaging (DSI) tractography of crossing fibers. NeuroImage 2008; 41: 1267–1277.

|  |
| --- |
